# Scaffold Generator: a Java library implementing molecular scaffold functionalities in the Chemistry Development Kit (CDK)

**DOI:** 10.1186/s13321-022-00656-x

**Published:** 2022-11-10

**Authors:** Jonas Schaub, Julian Zander, Achim Zielesny, Christoph Steinbeck

**Affiliations:** 1 Institute for Inorganic and Analytical Chemistry, Friedrich-Schiller-University Jena, Lessing Strasse 8, 07743 Jena, Germany; 2 Institute for Bioinformatics and Chemoinformatics, Westphalian University of Applied Sciences, August-Schmidt-Ring 10, 45665 Recklinghausen, Germany

**Keywords:** Cheminformatics, Chemistry Development Kit, CDK, Natural products, Scaffold, Scaffold tree, Scaffold network, Fragmentation, Chemical space, Clustering

## Abstract

The concept of molecular scaffolds as defining core structures of organic molecules is utilised in many areas of chemistry and cheminformatics, e.g. drug design, chemical classification, or the analysis of high-throughput screening data. Here, we present Scaffold Generator, a comprehensive open library for the generation, handling, and display of molecular scaffolds, scaffold trees and networks. The new library is based on the Chemistry Development Kit (CDK) and highly customisable through multiple settings, e.g. five different structural framework definitions are available. For display of scaffold hierarchies, the open GraphStream Java library is utilised. Performance snapshots with natural products (NP) from the COCONUT (COlleCtion of Open Natural prodUcTs) database and drug molecules from DrugBank are reported. The generation of a scaffold network from more than 450,000 NP can be achieved within a single day.

## Introduction

### Scaffold concept and applications

Molecular scaffolds, defined as the core structures of molecules and also referred to as chemotypes or frameworks in some studies, are a concept used in many areas of chemistry. In drug design, the scaffold of a molecule is considered the main structure that determines its shape and places the functional moieties into the right positions to interact with the target. For this reason, developing new drug molecules with different cores but similar biological activities has been termed “scaffold hopping” [1, 2]. Combinatorial chemistry makes use of the concept in designing compound libraries by substituting a set of scaffolds with combinations of different side chains. And structures in chemical patents are often defined analogously as Markush structures [3]. The intuitive chemical scaffold concept can also be utilised for classification purposes, especially in natural product (NP) research [4–7]. In cheminformatics, scaffold-based approaches can be applied for the analysis of high-throughput screening (HTS) data [6–10], mapping and visualising chemical spaces [5, 11], or even train-test splits of molecular data sets for machine learning projects [12].

Another application of scaffold-based methods is identifying privileged substructures in active molecules or NP that can be used as lead structures in the development of new drugs [5, 13–19]. Within NP chemical space, macrocyclic structures or cyclic peptides are of specific interest for these medicinal chemistry purposes [20–23].

### Scaffold approaches in cheminformatics

The first general definition of a molecular scaffold was the Murcko framework developed by Bemis and Murcko in 1996 [11]. According to this concept, a scaffold consists of all the rings in a molecule and the non-cyclic chains connecting them, called linkers. Excluded from the scaffold are all terminal side chains. In addition to the Murcko framework definition representing molecular properties like atomic elements and bond multiplicities, Bemis and Murcko introduced a more abstract representation that reduced each atom in the framework to a simple graph node and each bond to a simple graph vertex, called graph framework or archetype. The authors used their framework definitions to assess the structural diversity of a set of drug molecules.

In addition to ignoring all non-cyclic molecules, the Murcko framework has one major drawback: small changes in the ring structure or the addition of a cyclic substituent, e.g. a benzene ring in drug design or a sugar moiety in NP research, can lead to very similar molecules not being grouped together due to non-equivalent scaffolds. Therefore, multiple approaches have been developed for organising molecular scaffolds in a graph-based structure to relate similar scaffolds to each other and to create a systematic scaffold hierarchy [4, 5, 7, 9, 10, 13, 24–27].

Early work to this end was done by Xu and Johnson, who developed multiple concepts of dissecting Murcko frameworks into constituting ring systems or abstracting them into reduced representations. They used these concepts to assign molecular equivalence numbers to molecular structures and thus classify them within chemical libraries [25].

Wilkens et al. in their hierarchical scaffold clustering (HierS) approach [10] use a scaffold definition similar to Murcko frameworks but additionally include all atoms that are directly attached to rings and linkers via multiple bonds. Non-cyclic molecules are taken into consideration as well and are assigned scaffolds based on their multiple bonds. To build a scaffold hierarchy, the original scaffold extracted from each molecule is dissected into its smaller parent scaffolds first. This is done by generating all smaller scaffolds that can result from the stepwise removal of ring systems, i.e. isolated single rings or fused multiple rings that share bonds or atoms, from the original scaffold. After the removal of one ring, linker atoms that have become side chains are also removed. The process is finished when only the individual ring systems are left. Via a substructure search, the scaffold hierarchy is constructed in the second step by linking parent and child scaffold if the smaller parent scaffold is a substructure of the bigger child scaffold. In the end, a tree-like hierarchy results with the individual ring systems as roots at the top, and their combinations in more complex scaffolds on the following levels. A scaffold that is not a single ring system has multiple parents in the hierarchy.

While HierS overcomes most limitations of the Murcko framework approach and is a good first attempt for scaffold classification, it also has some disadvantages: ring systems are not split into their constituting single rings, which can be especially problematic when studying complex ring systems of NP where the approach may be too coarse-grained. In addition, child scaffolds are linked to multiple parents in the hierarchy, which is a multi-class assignment that is often undesirable for classification tasks.

The latter drawback of HierS is addressed in the structural classification of natural products (SCONP) approach by Koch et al. [5] that uses the same structural scaffold definition (apart from again ignoring linear molecules) but differs in its hierarchy construction routine. One major difference is that scaffolds are not dissected here. Only the directly extracted, original scaffolds of the studied molecule set are used to construct their relations in a tree-like fashion. A more complex scaffold is linked to only a single parent scaffold that is selected from all possible parent scaffolds representing substructures of the child following a set of chemical rules. These take characteristics of the parent scaffolds into account like hetero atom count, size, and frequency in the studied dataset. This last aspect makes the approach dataset-dependent, which can lead to problems in classification tasks.

A combination of scaffold dissection and single-parent assignments through chemical prioritisation rules is the scaffold tree approach by Schuffenhauer et al. [7]. As a first step, scaffolds are extracted from the given molecules according to the Murcko framework definition but additionally including all atoms connected via a double-bond to ring or linker atoms in the scaffolds. These elements are included as well to preserve correct hybridisation and structural alignment of the scaffold atoms. Via an iterative removal of rings, smaller parent scaffolds are created from the original child scaffolds. Ring perception for the removal is based on a smallest set of smallest rings (SSSR) approach. This way, ring systems sharing atoms or bonds between multiple rings are not considered as one entity but dissected into their constituting rings as well. One important aspect about the scaffold tree approach is the application of 13 chemical prioritisation rules at every ring removal step. Following these rules, only one terminal ring is specifically selected for removal and only one possible parent scaffold created at every scaffold dissection step. The term “terminal” indicates that the removal does not result in a disconnected scaffold structure. The specific prioritisation rules take only molecular characteristics of the rings, like size, hetero atom count, and aromaticity, into account and aim at removing the less characteristic, peripheral rings first to extract the characteristic, central parent scaffold. The scaffold dissection process continues until only one ring remains. When studying a collection of molecules, their original scaffolds and sets of created parent scaffolds are arranged in a hierarchy tree, the scaffold tree. Single-ring scaffolds form the roots and more complex scaffolds are placed at the higher levels. Due to the linear scaffold dissection process using the prioritisation rules, every child scaffold in the hierarchy is exclusively assigned to only one parent scaffold. Therefore, the scaffold tree represents a hierarchical, deterministic, and unique classification of chemical scaffolds. Unlike SCONP, it is dataset-independent because it does not consider the frequency of a scaffold in the studied collection. In conclusion, the scaffold tree is a useful tool for scaffold-based classification and visualisation of large compound sets and can be successfully employed to identify active scaffolds in HTS data and promising candidates for drug development [6–8, 28–30].

By definition of prioritisation rules, Schuffenhauer et al. intended to create a chemically intuitive classification system which opposes a classification focussing on pharmacophoric elements [7]. Also, its capability to identify biologically active substructural motives is limited because its exploration of possible parent scaffolds is limited due to the prioritisation rules. For this reason, Varin et al. introduced the concept of scaffold networks [9], where scaffolds are extracted and dissected analogously but without the application of prioritisation rules. In this way, every possible parent scaffold is generated for a given original scaffold and the resulting hierarchy, the scaffold Network, contains multi-parent relationships between its nodes. Varin et al. generated considerably more active scaffolds in primary screening data using scaffold networks compared to the scaffold tree approach. The reason for this is the exhaustive enumeration of parent scaffolds which leads to the scaffold network containing significantly more scaffolds than a scaffold tree. Additionally, a scaffold is not linked to all parent scaffolds that are substructures of it in the scaffold tree, only to the one determined as its characteristic core. As a consequence, this scaffold may be regarded to be less active.

The scaffold network approach explores the scaffold space more exhaustively and supports the identification of areas that a specific compound set does not cover. In addition, more virtual scaffolds can be identified, i.e. scaffolds that are only generated as a result of scaffold dissection and do not appear directly as original scaffolds in the given molecular structures. When studying a compound set linked to bioactivity data, these structures are usually of high interest when appearing frequently in active molecules [29, 30].

On the other hand, scaffold networks can become large and complex with a comparably small number of molecules, which makes it difficult to visualise them. When linked to bioactivity data, Varin et al. suggest to only include islands of relevant, active scaffolds in the display.

As a conclusion, scaffold trees are generally more suitable for a complete visualisation and overview of the defining motives and structural classes in a limited compound set. Whereas scaffold networks can be seen as more helpful for analysing compound sets linked with bioactivity data to reasonably limit the display and identify active substructural motives [9].

An even more extensive scaffold network approach was published recently by Manelfi et al. named “Molecular Anatomy” [26]. While the aforementioned approaches mostly rely on one single scaffold definition, respectively, nine different scaffold types of different abstraction levels were introduced here. All of them can be dissected analogously into parent scaffolds and linked in a network representation. This way, common substructure patterns can be identified on a higher abstraction level than with scaffold networks and more relevant similar compounds determined. This may be helpful for analysing HTS data or preparing structure activity relationship (SAR) studies of scaffolds and their side chains. However, this type of scaffold network including also more abstract scaffold representations has an even stronger tendency to grow very quickly with an increasing number of included structures and hence to quickly become unfathomable without sensibly limiting the display.

An analogous scaffold tree-like approach to hierarchical clustering based on Xu and Johnson’s more abstract scaffold representations was published by Medina-Franco et al. [24]. Here, scaffolds are not dissected into parent scaffolds but clustered in a tree structure based on scaffold representations with a lower chemical resolution at each higher level.

An inherently different approach to scaffold generation and clustering are methods based on analog series. Here, no a priori scaffold definition like the Murcko framework is applied. Instead, all structures in a given set are grouped into analog series based on methods like matched molecular pairs with additionally deriving precursor structures using the RECAP (Retrosynthetic Combinatorial Analysis Procedure) [31] rules. This way, the scaffolds extracted as representatives of an analog series take synthetic accessibility into account, an important aspect in medicinal chemistry but mostly ignored in the approaches above. The analog series and their representative scaffolds can be visualised by R-group tables, mapped into coordinate-based chemical space [32], annotated with activity information to support SAR studies, or used to extract favourable lead structures for drug design campaigns [17, 18, 33–38].

### Open implementations

Most of the original software tools implementing the scaffold-based approaches described in the previous section have not been published openly (Murcko frameworks, SCONP, scaffold tree) or are not findable anymore (HierS). As a result, a number of open re-implementations and more advanced, versatile software for scaffold analyses has been developed and published [28, 29, 39–44].

The first open software application that implemented a scaffold tree was Scaffold Hunter [28, 29, 39]. Starting as a tool mainly for generating and visualising scaffold trees, it has evolved into a multi-functional cheminformatics platform for visual data analysis. By default, the prioritisation rules are applied as published by Schuffenhauer et al. [7], but they can be customised by the user or even turned off completely. Varin et al. used the latter option to generate their scaffold networks using Scaffold Hunter [9]. The rich-client application is implemented in Java and employs the Chemistry Development Kit (CDK) [45–47] for cheminformatics tasks.

The open command-line tool Scaffold Network Generator [41] was designed to generate both, scaffold trees and scaffold networks. It lacks the extensive visualisation functionality of Scaffold Hunter but can therefore be integrated into automated analysis workflows that do not require human interaction. Scaffold Network Generator was implemented in Java as well and employs the CDK and Open Babel [48] cheminformatics toolkits. Unfortunately, it cannot be found at the internet address given in the original publication anymore.

The cheminformatics toolkit RDKit [49] recently integrated an extensive scaffold network functionality into its range of capabilities [40]. The module named “rdScaffoldNetwork” primarily offers the generation of scaffold networks based on a HierS-like scaffold dissection (no splitting of fused rings). Custom fragmentation rules can be added in the form of reaction SMARTS [50]. In addition, more abstract atom- and bond-generic scaffold representations can be generated. The new functionality has been employed in a study evaluating different approaches to automate chemical series classifications in medicinal chemistry [51].

These three open software tools for scaffold-based analyses are only a limited number of examples for many more such tools developed in the past years [42–44].

### Motivation

Structural scaffold analyses are relevant in diverse areas of cheminformatics, e.g. clustering, visualisation of chemical spaces, and SAR analyses [4–10, 26, 30]. Hence, numerous open software tools for such purposes have been developed [28, 29, 39–44]. The popular cheminformatics toolkit RDKit even integrated scaffold functionalities into its core modules. For the Chemistry Development Kit, only the generation of Murcko frameworks is currently available [52]. Outside core CDK, there is no open scaffold software library exclusively based on CDK to use in workflows and software based on the toolkit. Scaffold Hunter implemented its scaffold functionalities as part of a software application, and they cannot be easily extracted from it. Scaffold Network Generator is based on CDK but on Open Babel as well and not findable anymore.

Here, we present Scaffold Generator, an open, stand-alone Java library for scaffold functionalities based on CDK, to fill this void. It offers the generation of scaffold trees and scaffold networks with comprehensive additional scaffold-related functionalities. An integration into the main CDK modules is intended.

## Implementation

The Scaffold Generator library was implemented in Java version 17 and is based on the Chemistry Development Kit (CDK) version 2.8. The openly available source code can be found on GitHub: https://github.com/Julian-Z98/ScaffoldGenerator. With Scaffold Generator, different scaffold representations can be extracted from given molecules, dissected into parent scaffolds in multiple ways, and organised in scaffold trees and networks. These can be visualised using the GraphStream library version 2.0 [53, 54].

### Available functionalities

#### Scaffold types

**Fig. 1:**
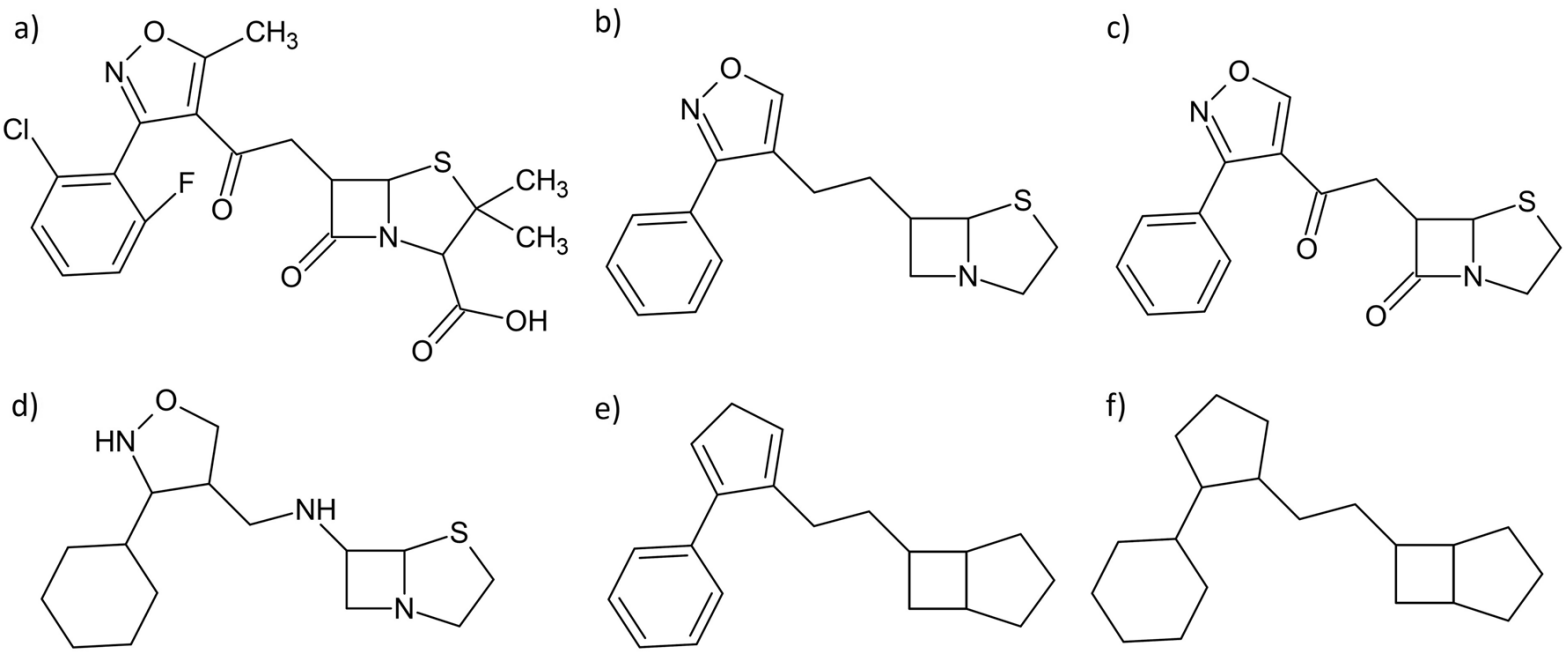
Different scaffold types available in Scaffold Generator. **A** Unaltered structure of the antibiotic agent flucloxacillin (PubChem CID 21319). **B** Murcko framework of flucloxacillin. **C** Scaffold of flucloxacillin. **D** Elemental wireframe of flucloxacillin. **E** Basic framework of flucloxacillin. **F** Basic wireframe of flucloxacillin

Molecules are passed to Scaffold Generator as instances implementing the central CDK molecular structure representation, the *IAtomContainer* interface [55]. From these, molecular scaffolds can be extracted according to different scaffold definitions available. These include the Murcko framework and the scaffold definition used in most of the established approaches, like HierS or the scaffold tree. It is based on Murcko frameworks but additionally includes all atoms connected to ring or linker atoms via double-bonds [7, 10]. In Scaffold Generator, this has been extended to all atoms connected via non-single bonds to cyclic or linker atoms. Higher bond orders than 2 are considered rare in such configurations but they influence the hybridisation and structural configuration of the scaffold as strongly as exocyclic or exolinker double-bonds. Another crucial aspect to consider here is the synthetic accessibility of the represented scaffolds that is significantly influenced by the presence or absence of exocyclic or exolinker multi-bonds. Additionally, two more abstract scaffold representations taken from Molecular Anatomy are available in Scaffold Generator: basic framework and basic wireframe [26]. Similar abstracted scaffold definitions have been described in earlier works as well, like the graph framework by Bemis and Murcko (analogous to basic wireframe) or the aryl cyclic system by Xu and Johnson (analogous to basic framework), but the naming was chosen here in analogy to Molecular Anatomy. A fifth scaffold type was analogously termed elemental wireframe. Here, all bonds are abstracted to single bonds, but hetero atoms are preserved (Fig. 1). For the creation of scaffolds of all types, the CDK *MurckoFragmenter* class [52] is used internally and the extracted Murcko framework is post-processed according to the chosen scaffold type if necessary. If a given molecular structure has no rings, no scaffold can be extracted and an empty *IAtomContainer* instance is returned.

**Fig. 2:**
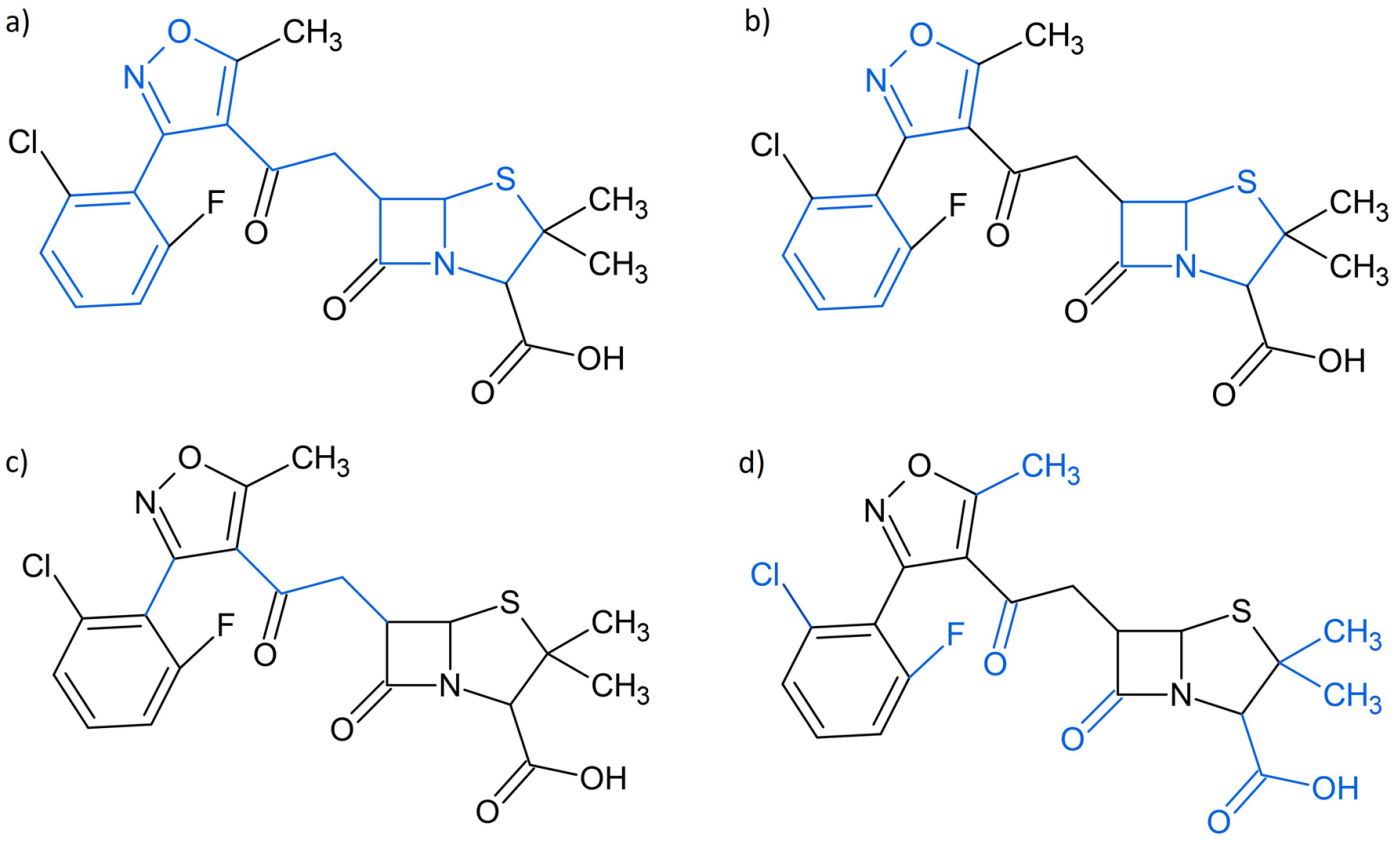
Dissection of scaffolds into building blocks. **A** Flucloxacillin with its Murcko framework marked in blue. **B** Rings of flucloxacillin marked in blue. It is important to note that the fused ring system on the right would be split into its two constituting rings in the structure set returned by the described routine of Scaffold Generator. **C** Linkers of flucloxacillin marked in blue. **D** Terminal side chains of flucloxacillin marked in blue

Another functionality of Scaffold Generator is to return the building blocks of scaffolds, i.e. rings and linkers, separately. The terminal side chains excluded from the scaffold structure can also be extracted (Fig. 2).

#### Ring detection

**Fig. 3:**
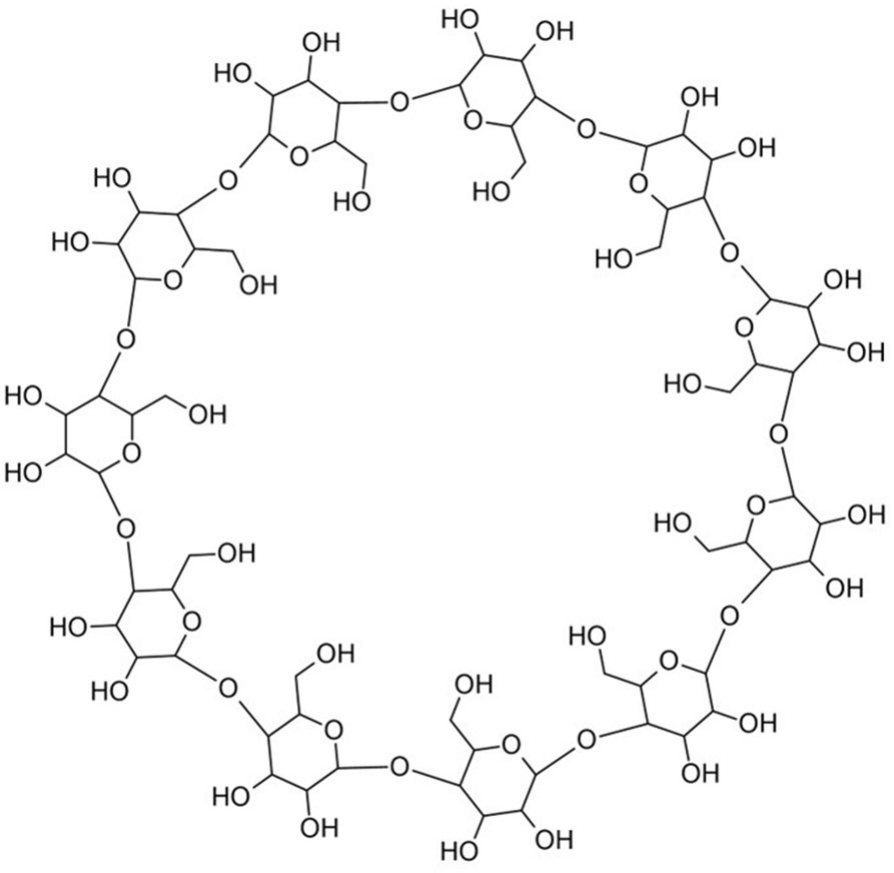
Rings of CNP0103752 taken from COCONUT. The CDK *Cycles.relevant* algorithm identifies 2059 rings here while *Cycles.mcb* detects 12

Scaffold Generator dissects fused ring systems, i.e. rings that share bonds or atoms, into their constituting separate rings. This is the case not only when returning scaffold building blocks but also for the generation of parent scaffolds (see below). Internally, the CDK *Cycles.relevant* cycle finder algorithm is employed for ring detection. This algorithm detects the logical union of all smallest sets of smallest rings (SSSR, also minimum cycle basis, MCB) in the given molecule [56, 57]. This way, fused ring systems are not detected as one entity, but their constituting cycles are detected separately. The *Cycles.relevant* cycle finder was chosen for Scaffold Generator to be in accordance with the original scaffold tree implementation [7]. But in rare cases, this cycle detection algorithm identifies too many rings in a given molecule, defined as more rings than there are atoms in the structure. One example is the natural product (NP) CNP0103752, taken from the COCONUT [58] database (Fig. 3). Since the overarching ring connecting all 11 glycosidic rings in the structure can be detected on many different paths, *Cycles.relevant* detects 2059 rings here. In cases like this, i.e. more rings are detected than there are atoms in the molecule, Scaffold Generator uses the algorithm *Cycles.mcb* instead, which identifies one single set of SSSR/MCB instead of the logical union of all possible ones [56, 57]. In CNP0103752, it detects a more useful number for this application of 12 cycles.

#### Ring removal

**Fig. 4:**
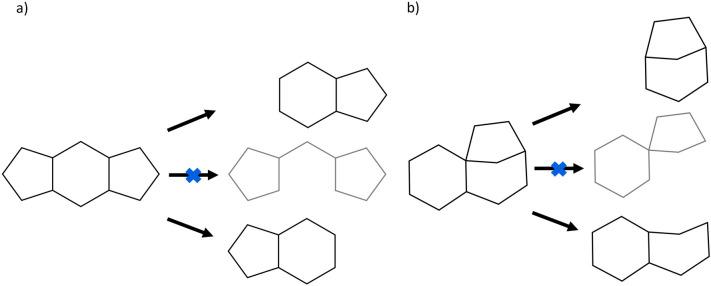
Impossible parent scaffolds in Scaffold Generator. **A** Dodecahydro-s-indacene (PubChem CID 13214318) representing an example scaffold cannot be dissected in a way that turns former ring atoms into linker atoms in the created parent scaffold. **B** Tricyclo[7.2.1.01,6]dodecane (PubChem CID 12,758,808) representing an example scaffold cannot be dissected in a way that creates a parent scaffold with a spiro-ring system which was not there in the molecule before

In the parent scaffold generation routines (see below), only rings adhering to a set of criteria are considered for removal at the individual dissection steps. The first requirement is that a ring needs to be terminal, i.e. its removal must not result in a disconnected scaffold structure. This is checked internally by removing all atoms and bonds constituting the respective ring from the scaffold, discarding potential side chains that were connected to it, e.g. when the scaffold structural definition is used, and assessing whether the structure does not consist of multiple disconnected parts afterwards. If it does, the ring in question is not deemed terminal and hence not removable. This routine of checking for terminal rings has two major consequences: Internal rings that could be removed without resulting in a disconnected structure by turning some of their atoms and bonds into linker structures are still not considered terminal (Fig. 4a). Secondly, the removal of rings from a scaffold cannot result in an artificially created spiro-ring system in Scaffold Generator (Fig. 4b). Such cases are described in the original scaffold tree publication [7] and the fifth prioritisation rule there is intended to prevent them if other rings can be removed first. But they are possible in general and would appear in a set of all possible parent scaffolds. Because the conversion of ring atoms to linker atoms and the artificial creation of spiro-ring systems are chemically non-intuitive when generating parent scaffolds, these possibilities have been excluded in Scaffold Generator.

Another requirement to consider a ring for removal is that it must contain at least one atom that is not part of another ring as well. This criterion is adopted from the original scaffold tree publication [7]. Here, the authors explain it with the example of adamantane. Using a ring detection algorithm that identifies the logical union of all SSSR in a structure, four rings are identified here and no atom is part of only one of them (compare Schuffenhauer et al. [7] Scheme 2). Hence, the removal of one ring is not possible because its atoms and bonds that are part of other rings as well are generally preserved in the Scaffold Generator ring removal routines. Structures like adamantane are therefore not dissected at all.

A similar case of structures that cannot be dissected are specific fused aromatic systems, i.e. aromatic rings that share the same atom with at least two other rings. When removing an aromatic ring sharing a bond with another ring, Scaffold Generator turns the shared bond into a double-bond to preserve the correct hybridisation of the formerly shared atoms in the remaining ring. In arrangements where the aromatic ring to remove shares an atom with at least two other rings, this double-bond insertion is not possible without violating valence rules. Such structures are not dissected as a consequence. This behaviour follows the ring removal algorithm described in the original scaffold tree publication (compare Schuffenhauer et al. [7] Scheme 3). But Scaffold Generator makes one addition here: In the original scaffold tree, this double-bond insertion is only done if an aromatic ring is fused to a non-aromatic ring and the aromatic ring is removed. In Scaffold Generator, it is also done if the remaining ring is aromatic as well. This addition has been made to preserve hybridisations and aromaticity in the remaining ring and to ensure that aromatic ring systems, if they can be dissected, are decomposed into parent scaffolds that can always be represented as valid contributing structures (as opposed to resonance hybrids). As a consequence, Scaffold Generator does not dissect most fused aromatic ring systems, e.g. pyrene. In these systems, most rings cannot be removed without altering hybridisations and bond orders in the remaining ones. And since a partial dissection does not appear reasonable because it would not produce meaningful parent scaffolds, these structures are not dissected at all. A possible future extension to Scaffold Generator could be a routine that extracts meaningful parent scaffolds from fused aromatic systems, e.g. a benzene ring as root scaffold from pyrene and similar structures.

**Fig. 5:**
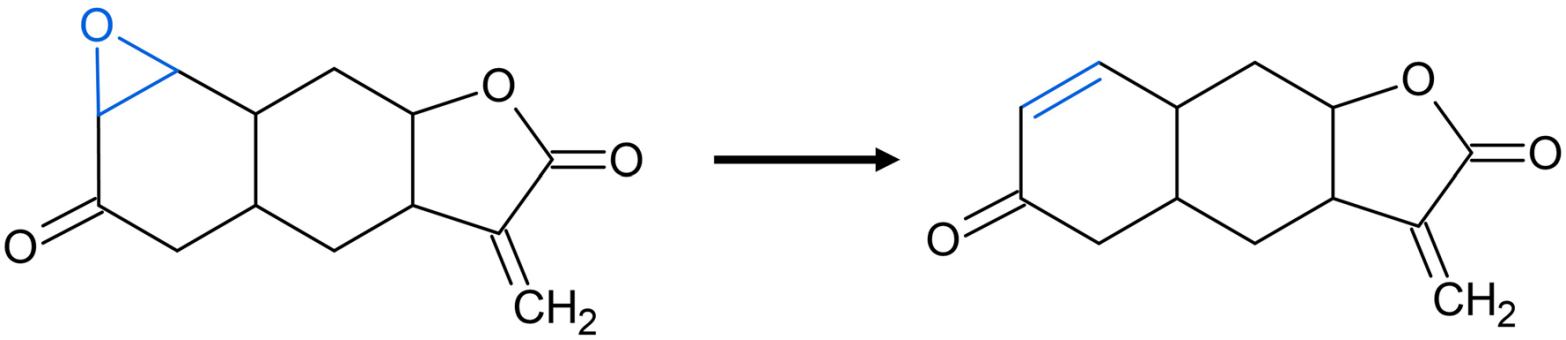
Removal of 3-membered hetero cycles. If the oxirane ring marked in blue is removed from himeyoshin (COCONUT CNP0151718) during parent scaffold generation, the bond shared with the cyclohexanone ring is turned into a double bond

Another specially treated system are rings of size three containing one hetero atom that share the bond opposite to the hetero atom with another ring (Fig. 5). When rings like this are removed, the shared bond is turned into a double bond to produce the precursor structure the hetero atom was most likely added to. This special case is described in the first ring removal prioritisation rule by Schuffenhauer et al. [7] but is part of the general ring removal routine of Scaffold Generator. This deviation from the original implementation does not influence the parent scaffold generation according to the scaffold tree prioritisation rules but is important to note for the enumerative generation of all possible parent scaffolds (see below).

#### Scaffold trees and networks

**Fig. 6:**
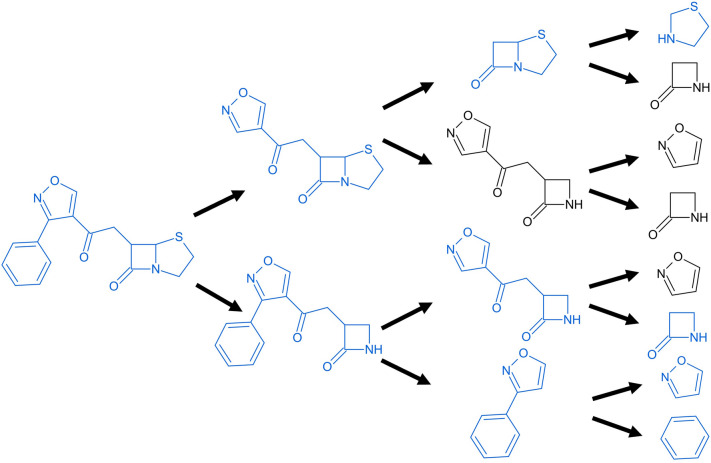
Enumerative parent scaffold generation of flucloxacillin. Conceptual depiction of the enumerative parent scaffold generation routine applied to the scaffold of flucloxacillin (on the left). All possible parent scaffolds that can be created through the removal of a terminal ring are created. Marked in blue are all structures that are returned by the routine, indicating that structures occurring multiple times are still returned only once

Using Scaffold Generator, extracted molecular scaffolds can be dissected in different ways. The first one, as described above, is to decompose it into the constituting building blocks, i.e. rings and linkers. Another option is the enumerative removal that generates all possible parent scaffolds. At every iteration step, each ring adhering to the criteria listed above is removed separately to produce the resulting parent scaffold. This is repeated until only single-ring scaffolds remain, or no ring is removable anymore. These final scaffolds are called the root scaffolds. All generated parent scaffolds are substructures of the original scaffold. An example for the enumerative removal is shown in Fig. 6. This routine can be applied to a given molecule and it returns a list with all possible parent scaffolds plus the original scaffold of the molecule. Parent scaffolds generated multiple times in the enumerative removal are returned only once. This scaffold dissection routine is the basis for generating scaffold networks. The dissection result of a single molecule can already be represented as a scaffold network by returning it as the corresponding data structure instead of a list.

**Fig. 7:**
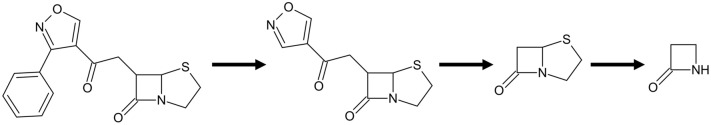
Schuffenhauer parent scaffold generation of flucloxacillin. Conceptual depiction of the parent scaffold generation routine employing the Schuffenhauer prioritisation rules applied to the scaffold of flucloxacillin (on the left). The rules are used to select only one parent scaffold out of all possible ones at every dissection step

Scaffold Generator implements the 13 chemical prioritisation rules that are applied in the original scaffold tree publication to specifically select only one parent scaffold at every scaffold dissection step [7]. In principle, these rules are applied to select only one ring removal path from all possible ones that are pursued in the enumerative removal (compare Fig. 6). Only a few minor changes have been done to the original rules and underlying routines as reported above. Additionally, the final tie-breaking rule has been adapted to use unique SMILES representations [59, 60] as produced by the CDK, instead of canonical ones. From a given molecular structure, Scaffold Generator can generate a list of all parent scaffolds resulting from the Schuffenhauer dissection routine, plus the original scaffold (Fig. 7). It produces the structures that can be used to build a scaffold tree in the second step. As with scaffold networks, a scaffold tree can already be constructed from a single molecule as well.

**Fig. 8:**
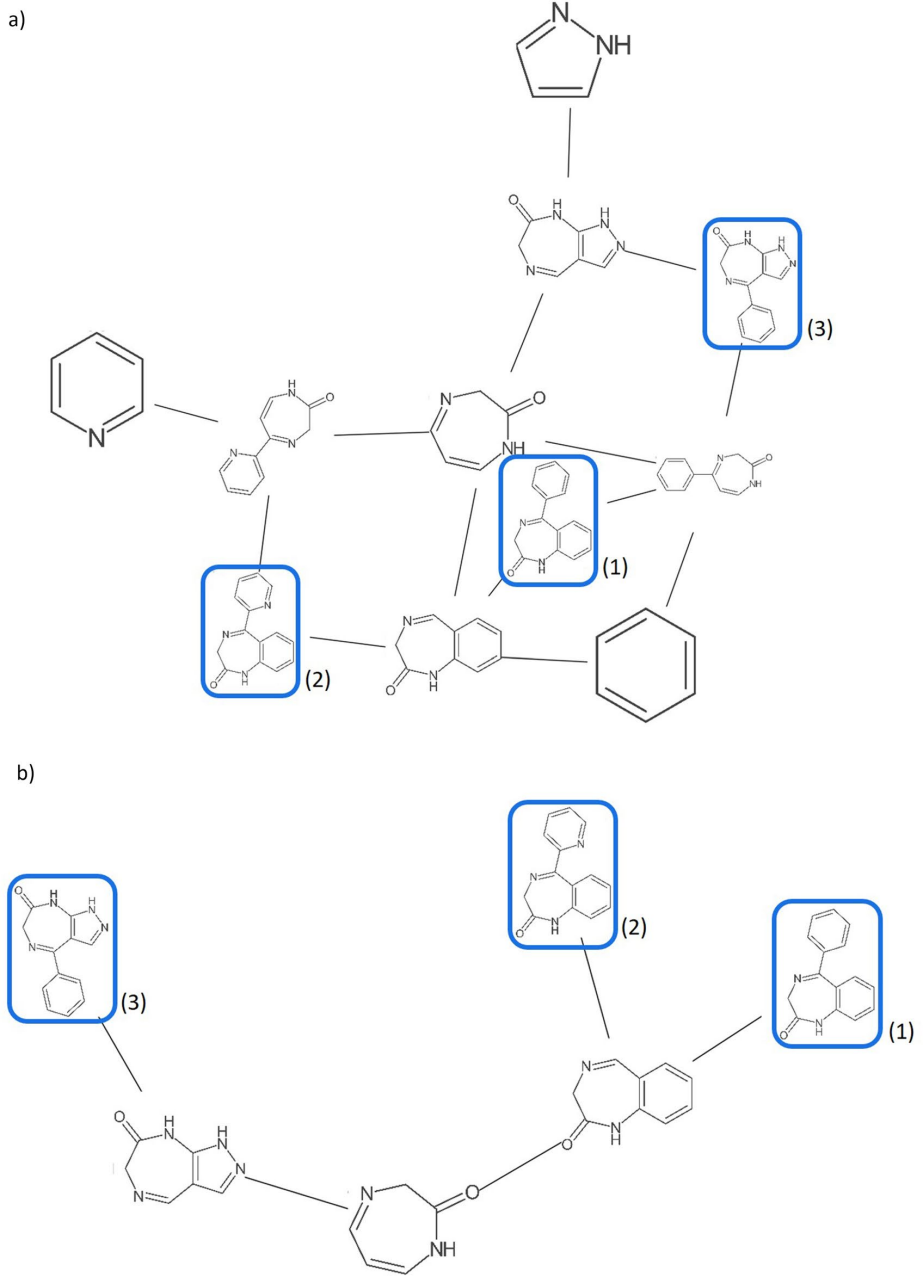
Scaffold network and tree depicted with the Scaffold Generator GraphStream visualisation. The scaffold network (**a**) and scaffold tree (**b**) of diazepam (PubChem CID 3016) (1), bromazepam (PubChem CID 2441) (2), and zolazepam (PubChem CID 35775) (3) are displayed side-by-side for direct comparison (original scaffolds marked in blue). All three compounds are diazepinenones, a class of anxiolytics. The scaffold tree correctly identifies the diazepinenone ring as root scaffold of all three structures. But the scaffold network additionally reveals that diazepam (1) shares two-ring parent scaffolds with both the other structures, respectively. It also shows that the benzene ring is shared by all three compounds as well

The main functionality of Scaffold Generator is the construction of scaffold trees and networks from given molecule collections (Fig. 8). In the first step, the first molecule in the given collection is dissected into its parent scaffolds and the result is used to build the starting point of the desired structure. One by one, the remaining molecules are decomposed as well and their original scaffolds and parent scaffolds added to the tree or network if they are not already part of it. Scaffold Generator implements data structures that manage the graph nodes representing scaffolds and their parent–child connections as edges in scaffold trees and networks. Both graphs are subdivided into levels with the root scaffolds on level 0 and their child scaffolds on the consecutive levels. The leaves are formed by the original scaffolds of the given molecules. But it is important to note that lower levels down to the roots can contain original scaffolds as well, e.g. when single-ring molecules are part of the given molecular set. The merging routines that are employed in the construction of a tree or network to add more scaffolds to it are also accessible after the final structures have been returned.

The scaffold tree and network structures differ in some aspects: In scaffold trees, each node has only one parent node. This results from the Schuffenhauer scaffold dissection where a scaffold produces only one parent scaffold in each step. In scaffold networks, on the other hand, a node can have multiple parents since a scaffold usually produces multiple parent scaffolds in each step during the enumerative removal.

Another distinct aspect of scaffold trees is that only those molecules with their original scaffolds and parent scaffolds can be combined in one tree that share the same root scaffold. This is the scaffold (usually a single-ring scaffold) which results as parent scaffold in the final step of the Schuffenhauer dissection. It is unambiguously determined by the prioritisation rules. Scaffold Generator compiles the generated scaffolds of multiple molecules in one scaffold tree instance if they have the same root scaffold. If molecules with different root scaffolds are given in the molecule set, multiple scaffold tree instances will be created and returned in a list, termed scaffold forest in the nomenclature of Scaffold Generator. In the construction of scaffold networks, only one parent scaffold, i.e. at least one ring, needs to be shared between two molecules to be able to combine them in one network. But the scaffold network data structure of Scaffold Generator is also able to handle multiple disconnected graphs of scaffolds in one instance, unlike the scaffold tree structure.

**Fig. 9:**
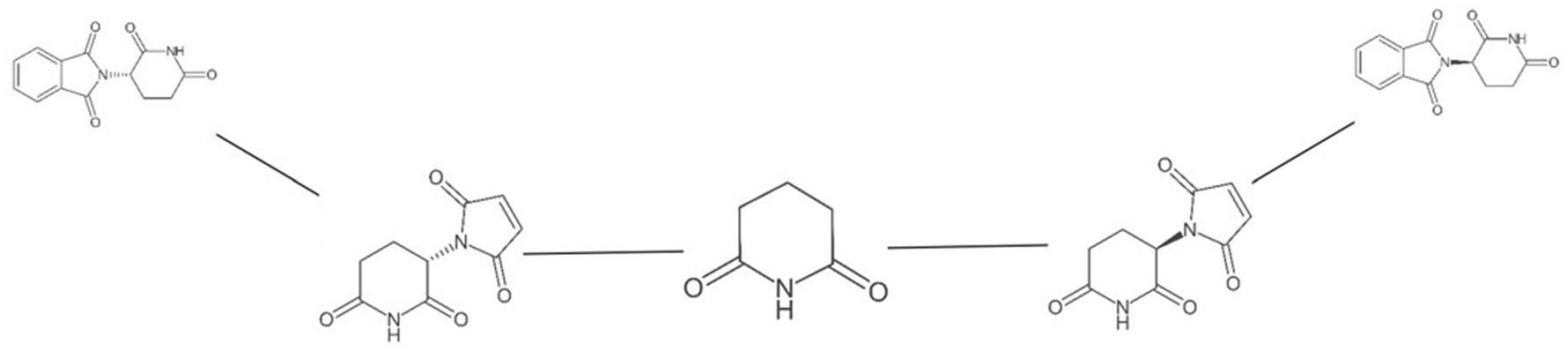
Scaffold tree with activated stereochemistry consideration. The Scaffold tree of ( +)-thalidomide (PubChem CID 75792, on the left) and (−)-thalidomide (PubChem CID 92142, on the right) with activated stereochemistry consideration is shown in the Scaffold Generator GraphStream display. If the consideration of stereochemistry in tree building was turned off, both compounds would be sharing the same two-ring scaffold as well

The tree and network data structures can generate an adjacency matrix representation of themselves that can be used for export or visualisation. Scaffold Generator offers an initial visualisation functionality for scaffold trees and networks based on the GraphStream library. The two structures can be visualised as graphs in a Java Swing application window. A layout algorithm attempts to place the nodes and edges as readable as possible but modifications to the layout can be done by dragging nodes. The display can also be zoomed and moved using key commands. Some figures in this publication have been created using the Scaffold Generator GraphStream display (Figs. 8 and 9). While this visualisation was helpful during the development process for visual inspection and debugging, it is not considered powerful enough for real-world use cases and will most likely not be part of a CDK integration of Scaffold Generator. A scaffold hierarchy visualisation tool that might sprout from Scaffold Generator as a separate project would have to be very interactive, i.e. zoomable, draggable, and collapsable. Especially scaffold networks tend to grow very fast with the number of included molecules. Therefore, their display needs to be limited in a comprehensive way, e.g. by only visualising islands of active scaffolds as proposed by Varin et al. [9]. Scaffold trees can become big as well, but they have the advantage that one can look at only one tree out of the forest at a time since they are disconnected.

When a tree or network is constructed, a crucial step is querying whether a scaffold is already part of it. This matching is done using SMILES representations of the scaffolds. The default setting is to use unique SMILES with aromaticity encoding but without stereochemical information. This can be adjusted, e.g. to include stereochemistry. Scaffold Generator generally retains given stereochemical information during scaffold creation and dissection by transferring the CDK *IStereoElement* [61] objects to the newly created structures. But this only works if all defining elements of a stereo group, i.e. atoms and/or bonds, are still present in the generated substructures. Since in the majority of cases side chains define stereochemistry and stereochemical information is often not given or incomplete in molecular data sets, the consideration of given stereochemical information in tree or network construction is turned off per default as stated above. But it can be enabled for use cases where it is relevant (Fig. 9). Other molecular characteristics that can generally be taken into account or not (depending on the specific use case) for the determination of equivalence between two structures in cheminformatic analyses are tautomeric forms or protonation states, for example. Standardising these structures if needed has to be done in a data curation protocol that is applied to the input structures before they are passed to Scaffold Generator.

The instances representing scaffold nodes in the trees and networks contain structural information about their scaffold and have references to their parents in the hierarchies. Additionally, they preserve SMILES codes of their origin molecules, i.e. structures from the data set that possess the respective scaffold. These origins are subdivided into virtual and non-virtual ones. Non-virtual origin molecules are those that have the node scaffold as their original scaffold, e.g. their Murcko framework. Virtual origins on the other hand are molecules that generate the respective scaffold only through enumerative or Schuffenhauer dissection, i.e. it is one of their parent scaffolds. This concept has been introduced in Scaffold Generator based on the definition of virtual scaffolds described in the literature [29, 30]. This term denotes scaffolds that are not directly in the data set but only identified when parent scaffolds are generated. If a scaffold node has only virtual origins, it is a virtual scaffold in Scaffold Generator. When analysing the results of a high-throughput screening (HTS) campaign, virtual scaffolds can be of particular interest if many of their child scaffolds exhibit bioactivity. A promising next step can be a second screening with a smaller library based on this scaffold because the first screen might have failed to include the true active scaffold structure.

An annotation of scaffold nodes in trees or networks with e.g. bioactivity data can be achieved via the stored origin molecules as well. One way to do this is to deposit the (unique) SMILES representation of the molecules in the studied data set linked to the respective annotation in a map structure. After the hierarchy is generated, its nodes can be annotated through comparing the origin molecule SMILES codes with the previously compiled annotation map. This way, e.g. scaffold nodes could be coloured according to bioactivity [7] or the hierarchy display limited to active scaffolds [9] in a more advanced visualisation tool as proposed above. During the development of Scaffold Generator, it was decided against keeping the original *IAtomContainer* instances with their structures and properties as origin references in favour of only their SMILES representations to reduce random-access memory (RAM) consumption.

### Aromaticity handling

Aromaticity information and detection is relevant in multiple Scaffold Generator functionalities. As stated above, when an aromatic ring is removed, bonds it shares with other rings are turned into double bonds in some cases to preserve hybridisations and aromaticity. Since this is not possible in all configurations, aromaticity information is also relevant in the determination of possibly removable rings (see above). And many fused aromatic ring systems, e.g. pyrene, are not dissected by Scaffold Generator as a result.

Aromaticity information is also significant in two of the 13 scaffold tree prioritisation rules for parent scaffold determination, namely rule 7 “A Fully Aromatic Ring System Must Not Be Dissected in a Way That the Resulting System Is Not Aromatic Any More”) and rule 11 “For Mixed Aromatic/Nonaromatic Ring Systems, Retain Nonaromatic Rings with Priority”) [7]. The seventh rule makes it necessary to generate all possible parent scaffolds producible by the removal of one ring at the given dissection step and apply aromaticity determination to each of them to assess whether aromaticity was lost in the remaining ring(s). Because this consumes a lot of computation time and aromaticity should be conserved in most cases through the double-bond insertion, the application of the seventh prioritisation rule can be turned off individually in Scaffold Generator.

Aromaticity determination in CDK and hence in Scaffold Generator is carried out by *Aromaticity* instances [62] constructed from the combination of an *ElectronDonation* model [63] and a *CycleFinder* algorithm [56]. The former defines which atom types can contribute how many electrons to the aromatic system and the latter determines the cycles that can form them. All aromaticity models in CDK loosely follow the Hückel rule heuristic [62]. The specific *Aromaticity* instance used in Scaffold Generator can be configured because different models are suited for different applications.

Since multiple intermediate steps in scaffold dissection rely on aromaticity information of specific substructures, an initial aromaticity detection is applied at the primary scaffold generation. And again at the end of a scaffold dissection process, a final aromaticity detection is applied to all generated parent scaffolds to make sure that the aromaticity information stored on the scaffold objects is in agreement with the returned structures. This last step might lead to cases where the same ring is not detected as aromatic in a smaller parent scaffold but in the bigger child scaffold in which it is a substructure. This is due to the cycle finder algorithms usually employed for aromaticity detection that are not SSSR-/MCB-based but also take cycles into account that span multiple rings of the molecule. It should be interpreted in the way that the ring in the parent scaffold gained aromaticity in the child scaffold through combination with other rings.

An additional option is to turn off aromaticity detection completely in all Scaffold Generator routines. This was implemented because this process takes a lot of time and makes the results of scaffold dissection routines dependent on mostly toolkit-specific and heuristic aromaticity models. If it is disabled, initially defined aromaticity information in the input structures is preserved.

It must also be noted here again that all aromaticity models in CDK are based on the Hückel rule, which is the most used heuristic for aromaticity determination but not the only one and has a long list of exemptions. Furthermore, it is only a heuristic determination method for the concept of aromaticity, which is itself not uniquely defined [64–67].

### Settings and options

The functionalities and routines of Scaffold Generator can be adopted for various applications by a variety of settings available (Table 1). Five different structural scaffold definitions can be chosen for initial scaffold extraction and scaffold dissection (Fig. 1). The default setting of the scaffold mode setting is to use the scaffold including all atoms directly connected to rings or linkers via non-single bonds.

**Table 1:** Settings and options of Scaffold Generator

Setting name	Options	Default
Scaffold mode	Scaffold	Scaffold
	Murcko framework	
	Basic wireframe	
	Basic framework	
	Elemental wireframe	
Determine aromaticity	True/False	True
Aromaticity model	All combinations of *CycleFinder* and *ElectronDonation* instances available in CDK	*ElectronDonation.cdk* and *Cycles.cdkAromaticSet*
Retain only hybridisations at aromatic bonds	True/False	False
Rule seven applied (“A Fully Aromatic Ring System Must Not Be Dissected in a Way That the Resulting System Is Not Aromatic Any More” [7])	True/False	True
SMILES generator	All *SmilesGenerator* configurations available in CDK	*SmiFlavor.Unique* and *SmiFlavor.UseAromaticSymbols*

The settings listed in this table together with their options and default values are available in Scaffold Generator to adjust its results to specific use cases

Multiple steps in scaffold dissection and the construction of Scaffold trees and networks require the testing for equivalence of molecular structures. These include the enumerative generation of all possible parent scaffolds to avoid duplicates and the identification of equivalent scaffolds when merging trees or networks. In Scaffold Generator, this is done using CDK unique SMILES codes. To allow the user the definition of structural features taken into account at these steps, e.g. stereochemistry, isotopes, or aromaticity, the CDK *SmilesGenerator* [68] instance employed can be set externally. By default, stereochemistry and atomic masses are not encoded but aromaticity is. The set *SmilesGenerator* instance is also used to create SMILES codes for origin molecules of a respective scaffold stored on nodes of scaffold trees and networks. It is important to note here that molecular characteristics of the input molecules and resulting (parent) scaffolds, like protonation states or tautomeric forms, are taken by Scaffold Generator “as is”, or rather as they are represented in the chosen SMILES encoding. The only exemption is the detection of aromatic systems which is done on input structures by default. Therefore, users have to take care of preprocessing their input data sets according to their specific needs, e.g. standardising tautomeric forms and protonation states in all input molecules, before using Scaffold Generator.

Another option is to exclude or include the Schuffenhauer prioritisation rule 7. This rule makes it necessary to apply aromaticity detection to different parent scaffolds created for testing purposes. This procedure is time-consuming and might not lead to a definite decision in favour of one specific parent scaffold in most cases. But by default, it is activated to be in accordance with the originally published scaffold tree implementation [7].

The aromaticity detection done in multiple steps of scaffold dissection (see above) can be configured by choosing which CDK aromaticity model is to be employed for this purpose. By default, aromaticity is determined using the *ElectronDonation.cdk* model and the *Cycles.cdkAromaticSet* cycle finder algorithm.

Additionally, aromaticity detection can be turned off completely in all routines to preserve initial aromaticity information of the input structures and make the results less dependent on specific aromaticity models. If this is the case, rule 7 is automatically excluded from the Schuffenhauer prioritisation rules as well.

**Fig. 10:**
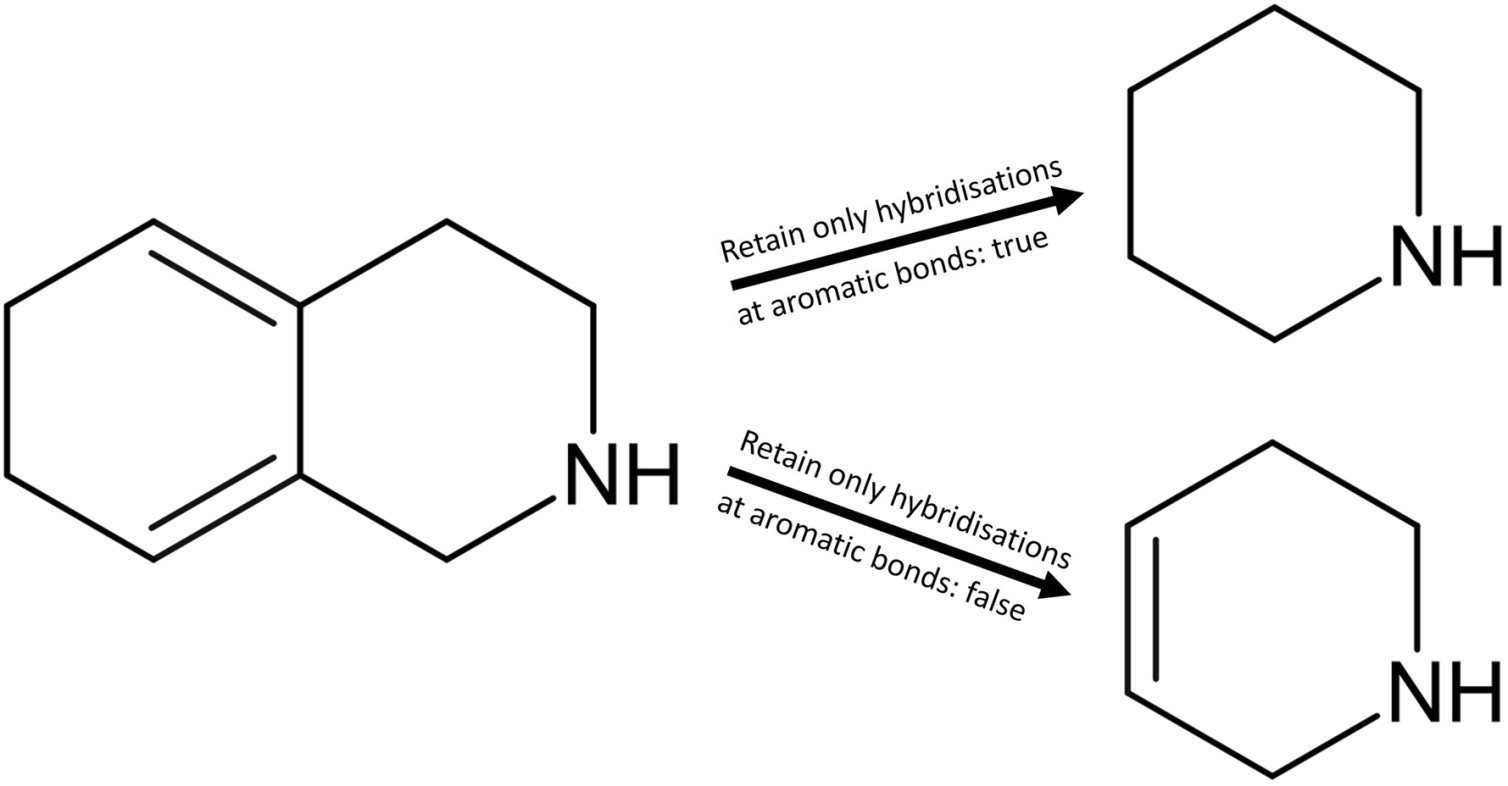
Parent scaffold of 1,2,3,4,6,7-hexahydroisoquinoline depending on the set value of the retain only hybridisations at aromatic bonds setting. When the cyclohexadiene ring is removed from 1,2,3,4,6,7-hexahydroisoquinoline (PubChem CID 89002720) in parent scaffold generation, the formerly shared bond with the piperidine ring is turned into a double bond if the retain only hybridisations at aromatic bonds setting is set to false. In this case, double bonds are always inserted if possible to preserve atom hybridisations in the remaining ring. If the setting is set to true, this is only done when an aromatic ring is removed. In this case, no double bond is inserted in the remaining piperidine ring

The fifth option of Scaffold Generator concerns post-processing after ring removal: As explained above, a double bond is inserted in some cases when an aromatic ring is removed to preserve hybridisation and aromaticity in the remaining ring(s) if possible. As an option, this insertion of double bonds can also be applied to non-aromatic systems wherever there are two sp^2^ hybridised atoms adjacent to a single bond that was previously shared between two rings. The bond is turned into a double bond if the two adjacent atoms would lose their sp^2^ hybridisation because of the ring removal and if it is possible without violating valence rules (Fig. 10).

### Software architecture

The central class of the Scaffold Generator library is *ScaffoldGenerator*. When instantiated, all available settings are set to their default values (Table 1) and can be adjusted using methods of the class. All main functionalities of Scaffold Generator described above can be accessed through an instance of the *ScaffoldGenerator* class, i.e. generation of scaffolds, their decomposition into building blocks, parent scaffold generation through enumerative or Schuffenhauer dissection, and the generation of scaffolds trees and networks. The two scaffold hierarchy structures are represented by a class of their own, respectively: *ScaffoldTree* and *ScaffoldNetwork*. Both extend the same base class, *ScaffoldNodeCollectionBase*, for basic functionalities and manage scaffold nodes as *TreeNode* or *NetworkNode* instances that both stem from the abstract base class *ScaffoldNodeBase*. These six classes manage scaffold structures, parent–child relationships of scaffold nodes, and origin molecule references. Trees and networks can be traversed and merged with instances of the same class, respectively. Scaffold trees can additionally be checked for validity, i.e. whether all nodes have parents, except the root node, and there is only one root node. Scaffold tree and network instances can also be exported as adjacency matrices along with scaffold structures for each represented node. This is utilised by the class *GraphStreamUtility* to display scaffold trees and networks in an interactive Java Swing application window with the GraphStream library.

The JUnit [69] test class *ScaffoldGeneratorTest* implements automatic tests for the basic Scaffold Generator routines, tests employing the GraphStream visualisation of scaffold trees and networks for visual inspection, and code examples for the application of Scaffold Generator. Another important set of test routines checks whether the Schuffenhauer prioritisation rules as implemented in Scaffold Generator are in accordance with the original implementation, based on the examples given in the scaffold tree publication [7]. Furthermore, the COCONUT database is used to test the basic routines on a large set of natural product (NP) structures.

The class *PerformanceTest* represents a command-line application based on Scaffold Generator that can be used to assess its computational speed on a given structure data file (SDF). The results on COCONUT and DrugBank [70, 71] are presented in the “Results and discussion” section.

## Results and discussion

A programming library for molecular scaffold functionalities named Scaffold Generator was implemented based on the Chemistry Development Kit (CDK). The openly available source code of Scaffold Generator can be found on GitHub: https://github.com/Julian-Z98/ScaffoldGenerator. It can be utilised to extract different types of scaffolds from input molecules and dissect them further into parent scaffolds using an enumerative generation of all possible ones or a dissection according to the scaffold tree prioritisation rules. Additionally, the scaffolds and parent scaffolds can be arranged in scaffold trees and networks with these hierarchies being visualised.

### Performance

Scaffold Generator can be packaged in a JAR file and used as a command-line application. It requires an SD file as input parameter and creates a performance snapshot of the main functionalities of Scaffold Generator with the given data set. First, all molecules are imported and stored in memory. From these, all structures having more than ten rings are discarded. This is done because they occur rather rarely but would influence the overall processing time disproportionally. No further filtering or preprocessing, e.g. removal of counter-ions or elimination of duplicates, is done for the purpose of this performance snapshot and the following exemplary analyses. For an initial performance snapshot, all remaining molecules are processed according to the enumerative generation of parent scaffolds and the parent scaffold generation according to the scaffold tree prioritisation rules. Afterwards, the dataset is subdivided into equally large portions. The total number of fractions has to be specified in the second command-line parameter. In each following step, a growing number of created molecule subsets is combined and all included structures used to build a scaffold network and a scaffold forest, i.e. a set of scaffold trees. The number of molecules and the needed processing time is logged in every step. In the final step, all scaffolds in the network and the trees, respectively, and their frequencies determined based on their numbers of origin molecules are exported to an output file. The scaffold structures are exported as SMILES strings.

For this article, two performance snapshots were conducted. The first one was done on the DrugBank database containing drug molecules (DrugBank “all structures” downloaded on 8th November 2021). For comparison, the COCONUT NP database (downloaded on 1st December 2021) was analysed as well. Additionally, for some analyses, a subset of COCONUT containing 40,000 structures was compiled from the complete collection using the RDKit MaxMin algorithm implementation [49, 72]. All analyses were conducted on a workstation computer with an Intel(R) Xeon(R) Gold 6254 CPU (18 cores, 3.10 GHz) and 512 GB RAM on a single core only (no multi-core parallelization). All Scaffold Generator settings were set to their default values.

The complete COCONUT database contained 406,747 NP structures (Table 2). 11,297 of these possessed 11 or more rings and were filtered. The remaining 395,450 NP were subjected to the parent scaffold generation according to the Schuffenhauer rules, which took 1,211,063 ms (20 min). On average, the dissection of one COCONUT NP into its scaffold and parent scaffolds according to the Schuffenhauer prioritisation rules took 3 ms. Generating all possible parent scaffolds with the enumerative routine took 2,037,357 ms (34 min) for the same molecule set. This is 5 ms per molecule on average.

**Table 2:** Performance snapshot of the mere parent scaffold generation routines applied to COCONUT and DrugBank

	COCONUT	DrugBank
Initial number of molecules	406,747	11,172
Number of molecules after filtering (< 11 rings)	395,450	11,127
Schuffenhauer dissection total	1,211,063 ms (20 min)	27,656 ms (0.46 min)
Schuffenhauer dissection average per molecule	3 ms	2.5 ms
Enumerative dissection total	2,037,357 ms (34 min)	33,938 ms (0.57 min)
Enumerative dissection average per molecule	5 ms	3 ms

The DrugBank data set of 11,172 molecules contained 45 structures with more than 10 rings that needed to be filtered. The Schuffenhauer dissection of all structures took 27,656 ms (0.46 min, 2.5 ms per molecule on average) and the enumerative parent scaffold generation took 33,938 ms (0.57 min, 3 ms per molecule on average).

It is interesting to note that the enumeration of all possible parent scaffolds at every step required more computation time than the application of up to 13 prioritisation rules at every step. This was the case for NP as well as drug molecules which have less rings in general. The latter characteristic of drug molecules as opposed to NP is also considered the reason for the lower time it took on average to dissect the DrugBank structures. It must also be noted that these processes, the pure dissection of each molecule, scale linearly with the number of molecules and can be parallelised in multiple threads for further speed up.

**Fig. 11:**
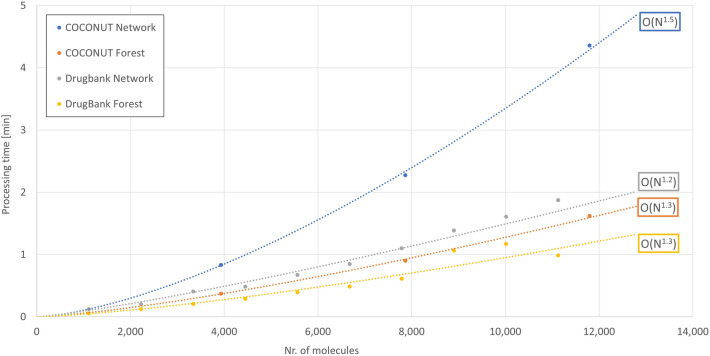
Performance snapshot of scaffold forest and scaffold network construction in DrugBank range of molecule number. The graph visualises the processing time it took to construct a scaffold forest or scaffold network depending on the number of input molecules taken from COCONUT or DrugBank. Exponential approximations have been applied to assess the scaling behaviour of the processes. The given range of the number of molecules is adjusted to the size of DrugBank (11,127 molecules)

**Fig. 12:**
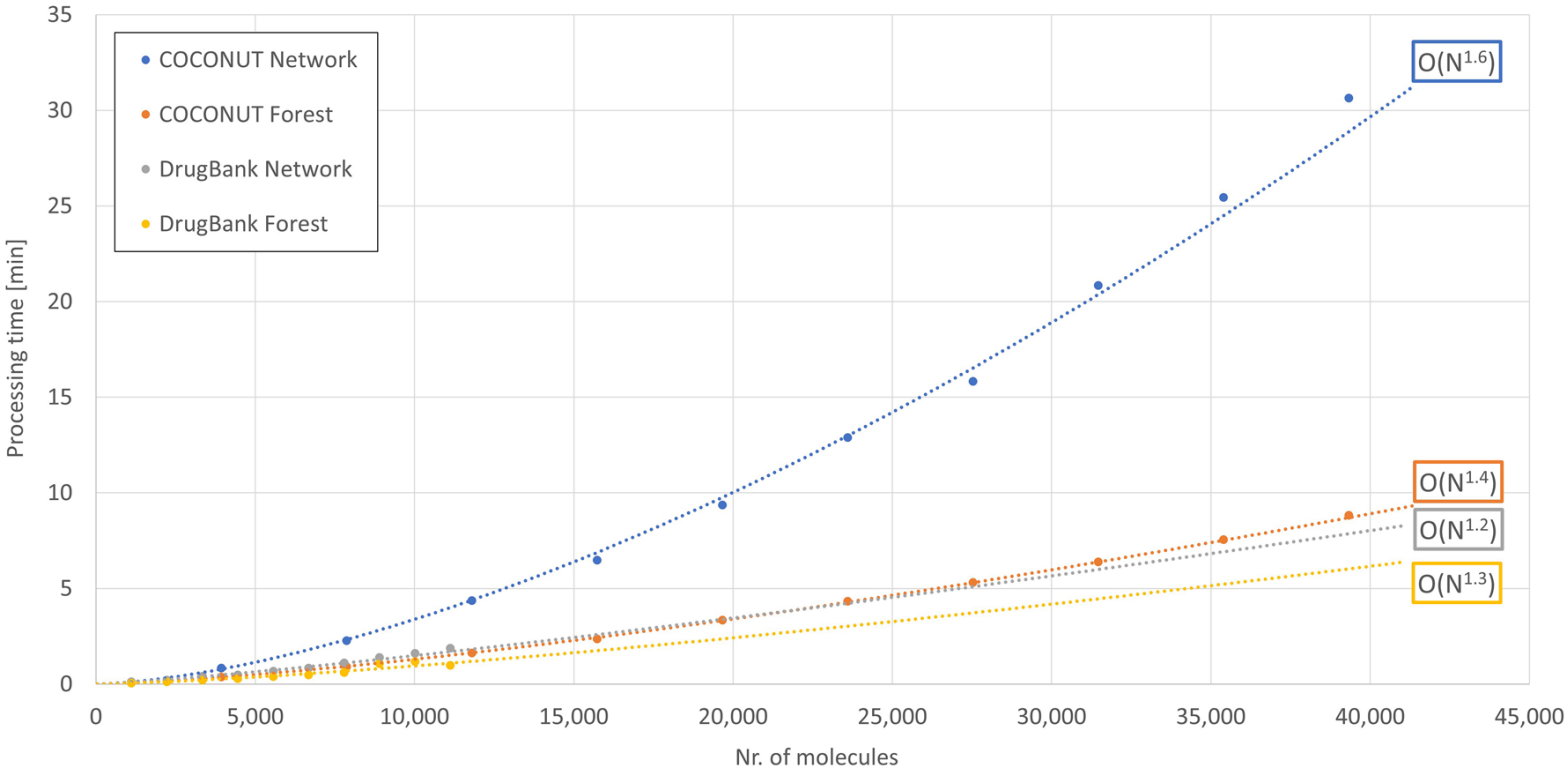
Performance snapshot of scaffold forest and scaffold network construction in COCONUT subset range of molecule number. The graph visualises the processing time it took to construct a scaffold forest or scaffold network depending on the number of input molecules taken from COCONUT or DrugBank. Exponential approximations have been applied to assess the scaling behaviour of the processes. The given range of the number of molecules is adjusted to the size of the curated COCONUT subset (39,324 molecules)

In a second step, it was measured how much time it took to construct scaffold forests and networks from an increasing number of molecules taken from the COCONUT subset and DrugBank, respectively. Figure 11 shows the results for the area of molecule number of DrugBank (0–11,127 molecules) and Fig. 12 for the area of the COCONUT subset (0–39,324 molecules). Exponential approximations show that the individual processes scaled between O(N^1.2^) and O(N^1.6^). This comparatively good scaling below a quadratic behaviour is most likely due to the stepwise construction of the scaffold hierarchies that repeats the two steps of scaffold dissection and integration for each molecule instead of generating all scaffolds first and constructing the hierarchy later using substructure searches to establish parent–child scaffold relationships.

Both, the generation of scaffold networks and trees from NP, scaled with higher exponents than the analogous processes for drug molecules, which can again be explained by the generally higher number of rings in the former class of compounds.

The generation of scaffold networks from NP structures scaled with the highest exponent. Since the number of scaffolds in a network grows faster than in a forest because more parent scaffolds are constructed for each molecule, it takes more time in network construction to integrate new molecules, i.e. their scaffolds. This traversal of the scaffold forest or network for the integration of new scaffolds is considered to be the algorithm step that dictates the scaling behaviour. In addition, this step would be more challenging to parallelise and speed up through multithreading because the same data structure would be accessed by all threads. The scaffold tree and network representations in Scaffold Generator are currently not implemented to be thread-safe, i.e. safe to use for concurrent modification.

According to the exponential approximation for the COCONUT subset of 40,000 NP structures, a scaffold network of up to 456,000 NP molecules could still be constructed in a single day using Scaffold Generator. The measured runtime for the complete COCONUT database of 395,450 compounds with less than 11 rings was 16.5 h (5 h for the construction of a scaffold forest). This is below the runtime of 19.2 h expected for this data set size according to the exponential function approximating the scaling behaviour of the COCONUT subset network generation. The underlying effect can be that with growing size of the network, less new scaffolds need to be integrated per newly added molecule. Here, one also has to take into account that the subset used for the performance and scaling snapshot was compiled using a diversity-preserving method [72]. This may have increased the effect even further.

The memory consumption of the scaffold tree and scaffold network constructed from the complete COCONUT database was below the 512 GB RAM available at all times but similar experiments on a machine with 256 GB failed.

### Most frequent scaffolds in COCONUT and DrugBank

The Scaffold Generator command-line application logs the numbers of different scaffolds in network and forest built from the given data set and exports the scaffolds as SMILES representations with their frequencies as a final step. These scaffold numbers for COCONUT and DrugBank can be found in Table 3. The COCONUT scaffold network contained 392,888 different (parent) scaffolds, while the DrugBank network contained 23,765. The COCONUT scaffold forest consisted of only 173,526 scaffolds distributed among 6200 individual scaffold trees. For DrugBank, it was 10,716 scaffolds in 766 trees. According to these numbers, the enumerative parent scaffold generation produced more than twice as many scaffolds as the Schuffenhauer dissection. Using a classification by root scaffolds, the two data sets could be classified into a number of different classes according to the number of resulting scaffold trees.

**Table 3:** Numbers of resulting scaffolds in scaffold network and scaffold forest constructed from COCONUT and DrugBank

	COCONUT	DrugBank
Number of molecules after filtering (< 11 rings)	395,450	11,127
Number of scaffold network scaffolds	392,888	23,765
Number of scaffold trees	6200	766
Number of scaffold tree scaffolds	173,526	10,716

**Fig. 13:**
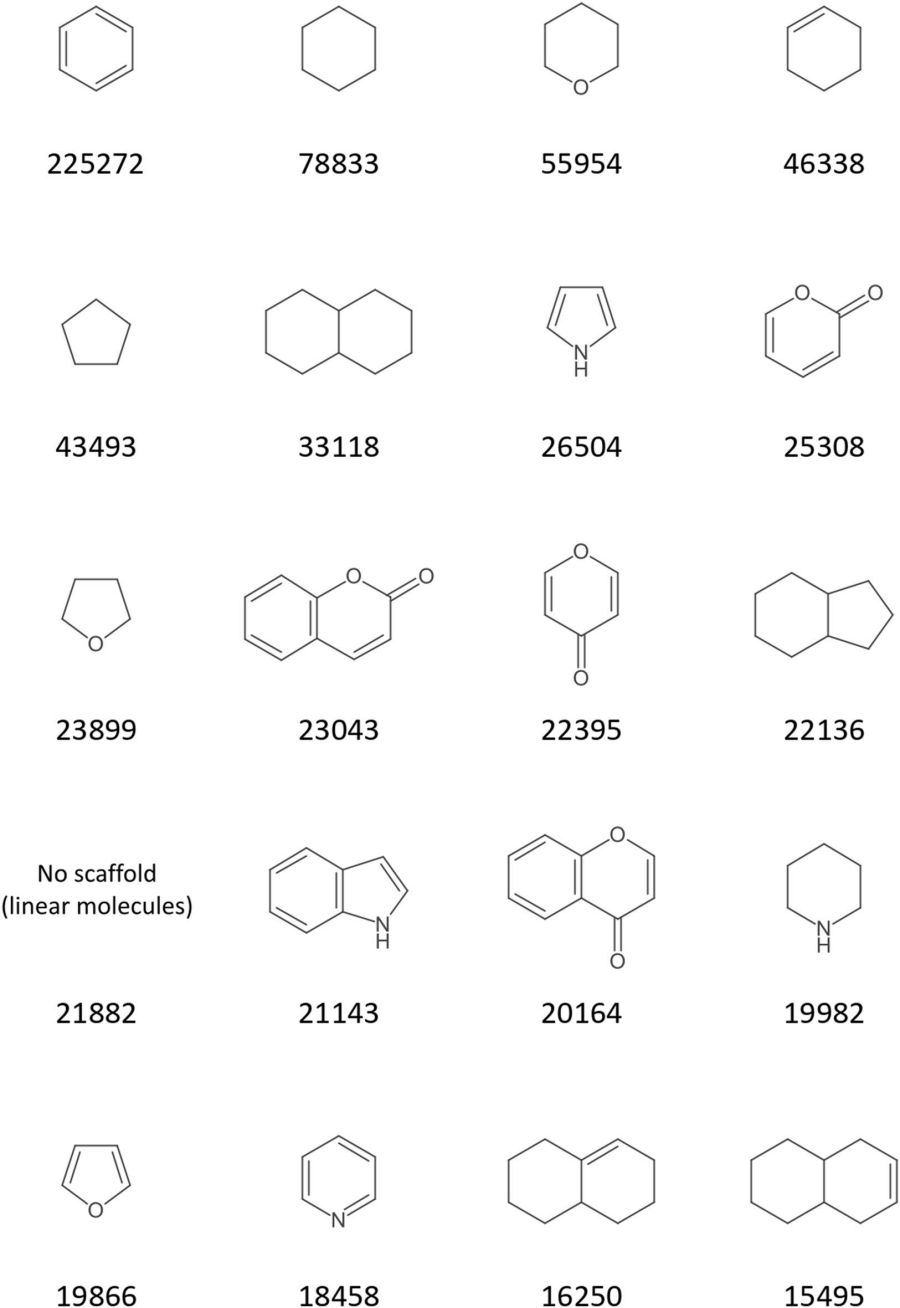
20 most frequent scaffold network scaffolds of COCONUT with their numbers of origin molecules

**Fig. 14:**
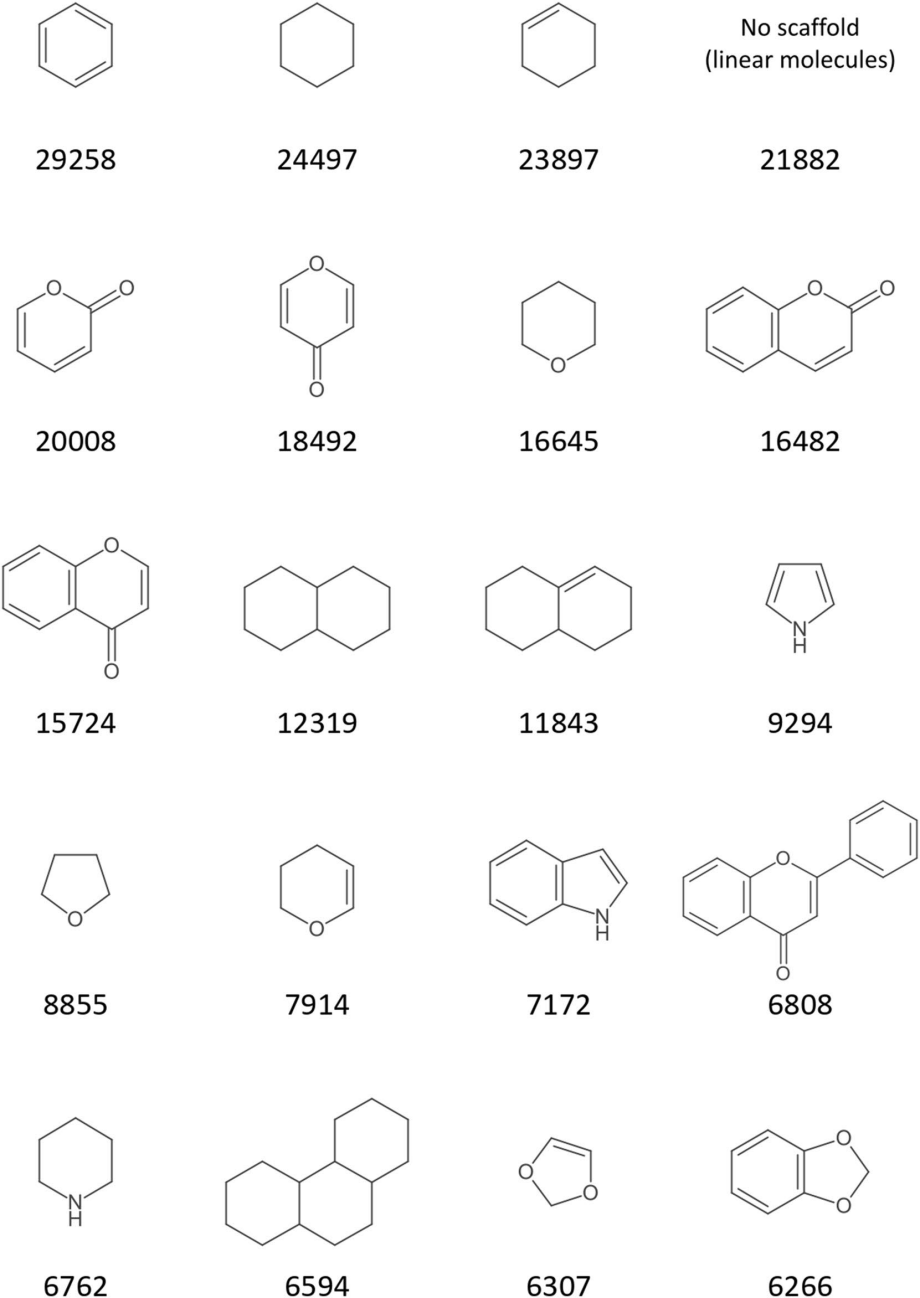
20 most frequent scaffold forest scaffolds of COCONUT with their numbers of origin molecules

The 20 most frequent scaffolds in the COCONUT scaffold network and scaffold forest, respectively, as determined in this exemplary showcase analysis, are displayed in Figs. 13 and 14. The frequencies are given as numbers of origin molecules that produced the respective scaffold in parent scaffold generation or had it as an original scaffold. The frequencies for the network scaffolds correspond precisely to the number of molecules that possess the respective scaffold as a substructure, whereas the frequencies for the forest scaffolds correspond to the number of molecules that possess the scaffold as their most characteristic or central parent scaffold in one step of the Schuffenhauer dissection according to the prioritisation rules. Hence, 225,272 COCONUT molecules contain a benzene ring (Fig. 13) but only in 29,258 molecules, it is the characteristic or central parent scaffold (Fig. 14). Still, it is striking that the benzene ring is the most frequent root scaffold in the forest because some Schuffenhauer prioritisation rules explicitly assign a low relevance to it and favour its removal over that of other rings.

As could be expected, the first ranks in both charts are dominated by single-ring scaffolds, since they represent the final stage of scaffold dissection and have the most origin molecules, therefore. The first ranks are also dominated by 6-membered rings and parent scaffolds that are most likely resulting from the dissection of polyketides. The frequency of oxygen-containing scaffolds is higher than that of nitrogen, as can be expected for NP. The empty cells in both charts represent empty scaffolds, i.e. scaffolds of molecules that have no rings. Hence, 21,882 molecules in COCONUT do not possess any circular structures. Of 406,747, the share of linear molecules is low (5%), but one should keep in mind that these structures are usually completely neglected in ring-based analyses like most scaffold methods.

**Fig. 15:**
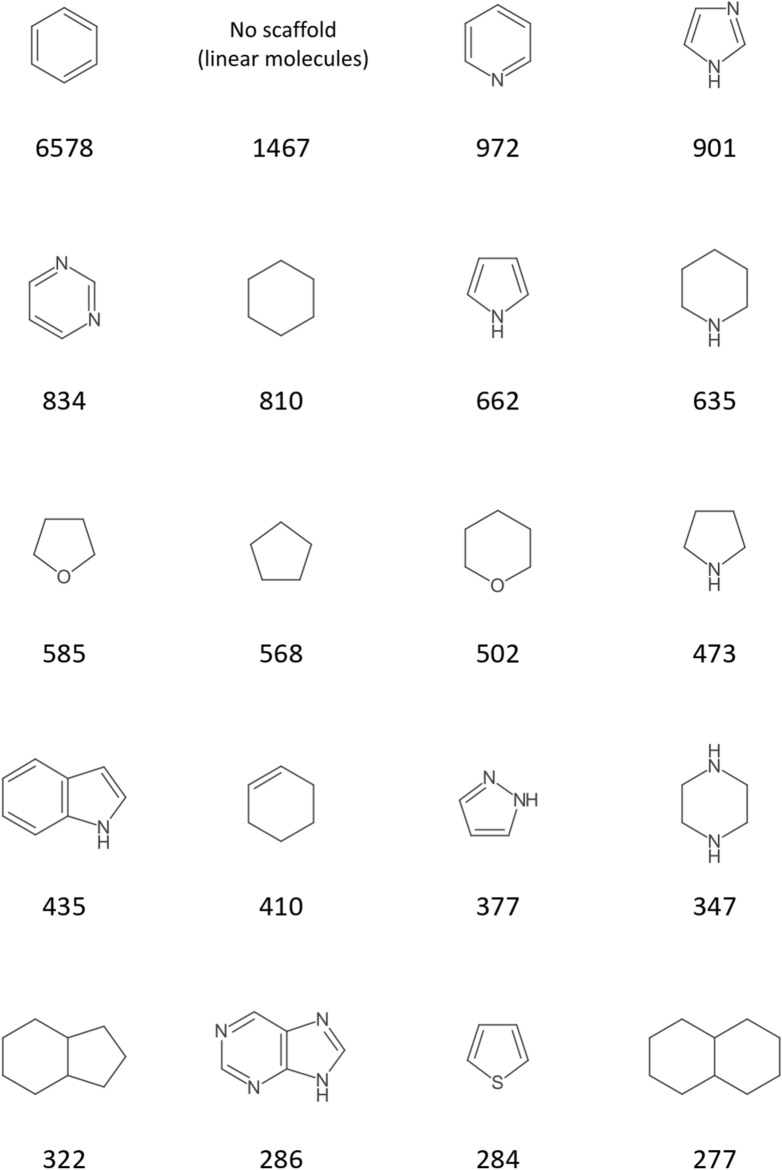
20 most frequent scaffold network scaffolds of DrugBank with their numbers of origin molecules

**Fig. 16:**
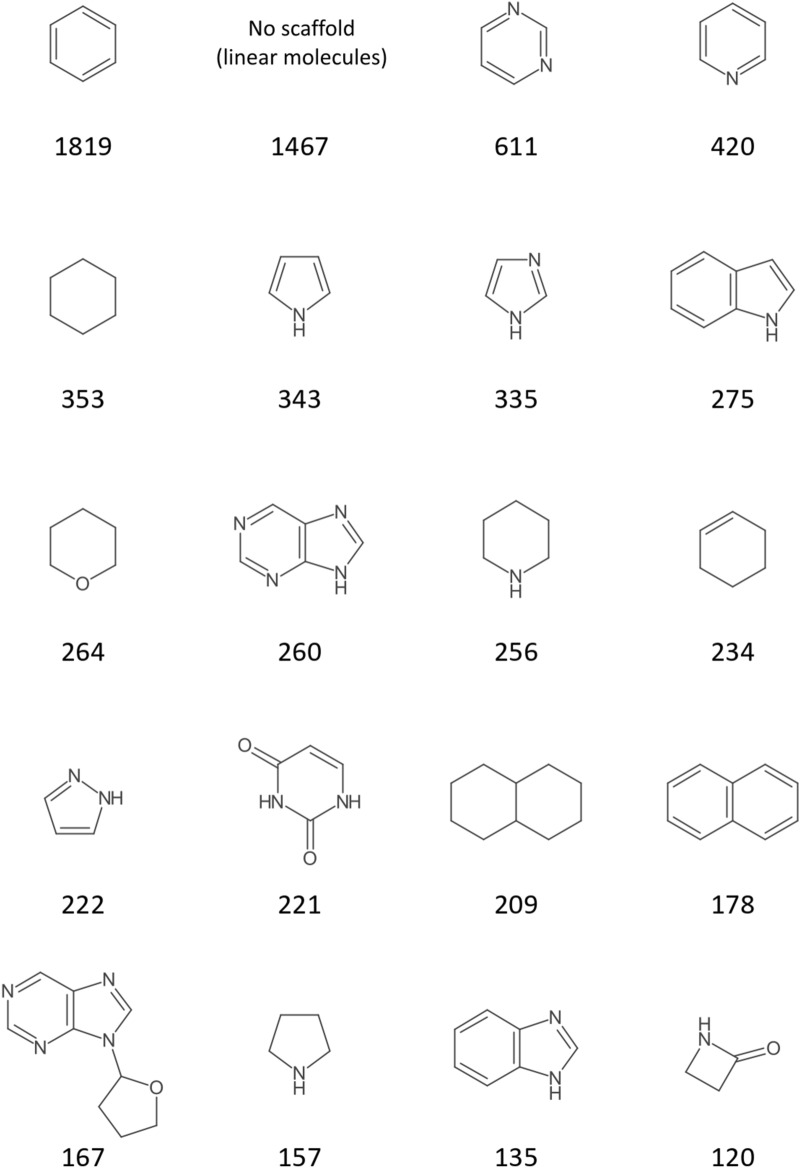
20 most frequent scaffold forest scaffolds of DrugBank with their numbers of origin molecules

Figures 15 and 16 analogously display the most frequent scaffolds of the created DrugBank scaffold network and scaffold forest. The first observation here is that the share of nitrogen hetero cycles is higher in these drug molecules than in NP structures. This has been reported before [73]. Also, the share of linear molecules (1467 of 11,172, 13%) is much higher than in NP. Benzene is again the most frequent scaffold in both analyses. But while it is by far the most frequent scaffold in the DrugBank network (6578 origin molecules compared to 972 for the second most frequent scaffold, pyridine), its prominence is way lower in the forest (1819 origin molecules compared to 611 for pyrimidine in second place).

The core results of this showcase analysis comparing the most frequent NP and drug molecule scaffolds (i.e. commonness of benzene, oxygen as the dominant hetero atom in NP, nitrogen in drug molecules) are in general agreement with similar studies [15, 19, 74–76]. A significantly higher prevalence of aromatic scaffolds in drug molecules as opposed to NP that most of these studies report cannot be observed here. This stresses that the results presented here are only a proof of concept for the application of Scaffold Generator. A more detailed analysis would first of all need an extensive data curation pipeline to standardise input molecules or filter or mark duplicates between the two data sets. Furthermore, a more extensive analysis of physicochemical property distributions in the extracted scaffolds could be conducted.

This analysis of the most frequent scaffolds in COCONUT and DrugBank is only supposed to serve as a basic example for what kind of studies Scaffold Generator may be used. These results may also have been achieved through the mere dissection of scaffolds into parent scaffolds and a subsequent matching and counting of the resulting structures. With its ability to generate and represent scaffold networks and forests, Scaffold Generator may be applied to a wider variety of analyses like hierarchical classification and clustering, chemical space mapping, or HTS data interpretation. But for these, a more powerful visualisation than the existing GraphStream-based one would be very helpful.

### Future work

Scaffold Generator meets the need for an open, versatile, CDK-based library for scaffold functionalities that can be employed in software and workflows built upon this cheminformatics toolkit. To make it more accessible to potential users, an integration into the CDK core modules would be desirable since the toolkit would benefit from having more scaffold functionalities available. A corresponding request to the library maintainers has been made.

Another aspect that would make Scaffold Generator more applicable is a more powerful visualisation functionality than the currently available one based on the GraphStream library. It should display the hierarchies in suitable layouts, i.e. a tree layout for scaffold trees and a similar layout for scaffold networks that arranges the network in its defined levels. The display should be draggable, zoomable, and collapsable. The latter aspect is especially important for scaffold networks that tend to grow very fast with the number of included molecules. For example, all scaffolds below a chosen node should be easily collapsable or only active islands of scaffolds should be displayed when bioactivity data is linked to the given molecules [9]. Especially the analysis of HTS data or the derivation of SAR insights would benefit from a versatile scaffold hierarchy visualisation. To further support these analyses, methods to display scaffolds and their parent scaffolds hierarchically in a standardised, directly visually accessible way, like the work of Alex M. Clark [77], should be explored in future developments.

Scaffold Generator can serve as core for a variety of scaffold-based functionalities. Classification, clustering, and scaffold-based fingerprints are possible applications that can be used in a second step for picking diverse training and test sets for machine learning models for example [12]. The concept of scaffolds and parent scaffolds as characteristic molecular fragments of molecules can help in the development of QSAR/QSPR models or computer-assisted structure elucidation. Applied to NP, scaffolds can serve as starting points for the creation of pseudo-NP that are regarded as promising candidates for new drug molecules [78, 79]. Additionally, the study of macrocyclic structures in NP with existing scaffold methodologies and the development of new, specialised approaches for these structures are promising ways of identifying new drug candidates [20–23].

Possible functional extensions of Scaffold Generator include the incorporation of more abstract scaffold representations, based on the work by Xu and Johnson [25], and the possibility to build scaffold networks or trees encompassing multiple scaffold definitions of varying chemical resolution, like in Molecular Anatomy [26] or the tree-like classification of Medina-Franco et al. [24]. A major addition to the functionality of Scaffold Generator would be the inclusion of analog series based scaffold methodologies. Since these have demonstrated significant relevance in the past years, this addition must be considered.

## Conclusion

An open, CDK-based, stand-alone Java library named Scaffold Generator has been developed to meet the need for scaffold functionalities in CDK-based workflows and software. It offers the extraction of different scaffolds, the dissection of scaffolds into building blocks, and the generation of parent scaffolds in two different ways. An enumerative parent scaffold generation routine produces all parent scaffolds that can be created through the removal of terminal rings and forms the basis for scaffold networks. Alternatively, only characteristic or central parent scaffolds can be extracted according to the Schuffenhauer prioritisation rules that are used to build scaffold trees. Scaffold trees and networks can be internally represented as data structures and visualised in a basic display based on the GraphStream library. The generation of a scaffold network from more than 450,000 natural product structures can be achieved in a single day. A request for the integration of Scaffold Generator into the CDK core modules has been made and the process started. Scaffold Generator may serve as a starting point for diverse scaffold-based software tools, e.g. for clustering or fingerprint functionalities.

## Data Availability

Data and software are freely available under the LGPL v2.1 licence. The source code of Scaffold Generator is available on GitHub at https://github.com/Julian-Z98/ScaffoldGenerator Project name: Scaffold Generator. Project home page: https://github.com/Julian-Z98/ScaffoldGenerator. Current version: v1.0.3. DOI of archived current version: https://doi.org/10.5281/zenodo.7245473. Operating system(s): Platform independent. Programming language: Java. Other requirements: Java v17 or higher, Maven v4 or higher, CDK v2.8 (fetched by Maven), GraphStream v2.0 (fetched by Maven), JUnit v4.13.2 (fetched by Maven). Licence: GNU Lesser General Public Licence (LGPL) v2.1. Any restrictions to use by non-academics: None

## Abbreviations

CDK: Chemistry Development Kit
CID: Compound IDentifier
CNP: COCONUT Natural Product
COCONUT: COlleCtion of Open Natural prodUcTs
CPU: Central Processing Unit
HierS: Hierarchical Scaffold clustering
HTS: High-Throughput Screening
JAR: Java ARchive
MCB: Minimum Cycle Basis
NP: Natural Product(s)
QSAR/QSPR: Quantitative Structure Activity/Property Relationship
R: Registered trademark
RAM: Random-Access Memory
RECAP: REtrosynthetic Combinatorial Analysis Procedure
SAR: Structure Activity Relationship
SCONP: Structural Classification Of Natural Products
SD(F): Structure Data (File)
SMARTS: SMILES Arbitrary Target Specification
SMILES: Simplified Molecular Line Entry System
SSSR: Smallest Set of Smallest Rings
